# Eosinophilic Oesophagitis Beyond the Oesophagus: High Prevalence of Chronic Rhinosinusitis‐Type Symptoms and Their Impact on Symptom Severity in Swiss EoE Cohort Study Patients

**DOI:** 10.1111/apt.70819

**Published:** 2026-06-25

**Authors:** Aidan Gebhardt, Andrea Kreienbühl, Thomas Greuter, Christoph Schlag, Simon Nennstiel, Catherine Saner, Jean‐Benoit Rossel, Tina Mauthe, Christian M. Meerwein, Alain Schoepfer, Michael B. Soyka, Alex Straumann, Luc Biedermann, the Swiss EoE Cohort Study Group, Patrick Aepli, Luc Biedermann, Ruggero Biral, Carine Blanchard, Simon Buetikofer, Emanuel Burri, Joachim Diebold, Carolien Dietrich, Annett Franke, Michèle Gwerder, Thomas Greuter, Chantal Hasler, Wolfram Jochum, Tanay Kaymak, Andrea Kreienbühl, Katline Metzger‐Peter, Fritz Murray, Simon Nennstiel, Peter Netzer, Jan Hendrik Niess, Gabrielle Reichhart, Cristiana Quattropani, Elodie Ristorcelli, Jean‐Benoit Rossel, Ekaterina Safroneeva, Catherine Saner, Alain Schoepfer, Philip Schreiner, Christine Sempoux, Dagmar Simon, Hans‐Uwe Simon, Alex Straumann, Sven Trelle, Achim Weber, Niels Willi, Elina Wuethrich, Marcel Zwahlen, Michael Scharl, Marcin Wawrzyniak

**Affiliations:** ^1^ Department of Gastroenterology and Hepatology University Hospital Zurich Zurich Switzerland; ^2^ Department of Internal Medicine Cantonal Hospital Frauenfeld Frauenfeld Switzerland; ^3^ Department of Gastroenterology and Hepatology Spital Wetzikon Wetzikon Switzerland; ^4^ Department of Gastroenterology and Hepatology Centre Hospitalier Universitaire Vaudois (CHUV) and University of Lausanne Lausanne Switzerland; ^5^ Department of Clinical Research University of Bern Bern Switzerland; ^6^ Department of Otorhinolaryngology, Head and Neck Surgery University Hospital Zurich Zurich Switzerland

**Keywords:** atopic comorbidity, chronic rhinosinusitis, eosinophilic oesophagitis, epidemiology, type 2 inflammation

## Abstract

**Background:**

Both eosinophilic oesophagitis (EoE) and chronic rhinosinusitis (CRS) are type 2 inflammatory conditions sharing pathogenic mechanisms, therapeutic responses and atopic associations. Data on the prevalence of CRS in EoE patients is missing.

**Aims:**

Our aim was to assess the prevalence of CRS‐type symptoms in EoE patients, the quality of life and shared characteristics of patients with both diseases.

**Methods:**

We assessed the point prevalence of CRS in Swiss Eosinophilic Esophagitis Cohort Study patients using a validated screening questionnaire comprising four symptom‐based diagnostic criteria. CRS was defined based on symptoms only (definition A) and including self‐reported CRS diagnosis by a physician (definition B). Point prevalence was tested against population prevalence using two‐sided binomial tests. Secondary outcomes were compared between patients with and without CRS. Multivariable logistic regression models were built to adjust for confounders.

**Results:**

211 patients (54%) answered our questionnaire. Prevalence of CRS‐type symptoms was 21.8% (definition A) or 25.1% (definition B), compared to 8.71% in the general population (*p* < 0.0001). CRS status was associated with increased EoE symptom severity. This association persisted after adjustment in multivariable logistic models, most consistently under definition A. 25% reported that their CRS symptoms were more debilitating than their EoE symptoms.

**Conclusion:**

CRS‐type symptoms are two‐ to three times more prevalent in EoE patients than in the general population and substantially impact quality of life. Our findings indicate that CRS may be under‐diagnosed in EoE and support routine CRS screening in EoE patients and cross‐speciality collaboration, particularly as both conditions benefit from therapeutic strategies targeting type 2 inflammation.

## Graphical abstract

Among 211 prospectively followed Swiss patients with eosinophilic oesophagitis, chronic rhinosinusitis was two‐ to three‐fold more common than in the general population. It was associated with greater oesophageal symptom severity and, in a quarter of cases, was experienced by patients as more debilitating than the underlying oesophageal disease.

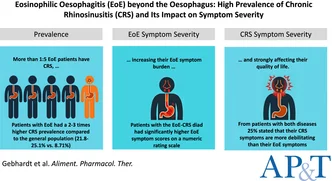

## 1 Introduction

It is increasingly recognised that eosinophilic oesophagitis (EoE) is associated with a systemic type 2 inflammation and respective atopic comorbidities, including atopic dermatitis, asthma and chronic rhinoconjunctivitis [1].

EoE prevalence was recently found to be around 143/100.000, of which approximately 63% were male [2]. The incidence seems to be rising, which is only partially attributable to increased awareness [3, 4, 5]. Nevertheless, diagnosis can be challenging, leading to diagnostic delay [6], elevating the risk of fibrostenosis in endoscopy [7]. A subset of patients first presents to otolaryngologists, where EoE remains commonly misdiagnosed despite expanding awareness [8].

Chronic rhinosinusitis (CRS) refers to a chronic inflammatory condition of the sinonasal mucosa [9]. A recent systematic review has found the global prevalence of CRS to be around 8.71% (95% CI, 6.69–11.33), albeit with high geographical variation [10, 11]. Patients with CRS present typically with nasal congestion, nasal discharge, altered sense of smell and facial pain. These four criteria form the basis of the validated screening criteria proposed by the 2020 European Position Paper on Rhinosinusitis and Nasal Polyps (EPOS) guidelines [12] and the clinical practice guidelines of the American Academy of Otolaryngology—Head and Neck Surgery (AAO‐HNS) [13, 14]. Formal diagnosis requires an endoscopy or CT scan, which also allows for the phenotype differentiation of CRS with or without nasal polyps.

Both diseases share several similarities, including the pattern of the immune‐mediated inflammatory response in the tissue, association with other atopic diseases, imperfect response to predominantly eosinophil‐depleting therapies (e.g., anti‐IL‐5 antibodies) [15, 16, 17] and successful clinical/endoscopic/histological response to topical steroids and, more recently Dupilumab [16, 18]. However, as both conditions are typically managed by organ‐specific specialists, neither EoE nor CRS may be on the radar of the reciprocal discipline or the general practitioner.

Recently, an 8‐fold higher prevalence of EoE was found in subjects with CRS, with even a 14‐fold increase in CRS patients with nasal polyps [19]. Yet, to the best of our knowledge, there is as of now no data on a respective reciprocal increase of CRS in EoE patients. A better understanding of CRS prevalence in EoE patients could facilitate earlier diagnosis, foster cross‐disciplinary management and ultimately improve quality of life.

We hypothesised that CRS has a substantially higher prevalence in patients with EoE compared to the reported prevalence in the general population and that there are differences in patient characteristics as well as disease burden in EoE patients with vs. without CRS.

## 2 Methods

This study had a hybrid design combining retrospective database analysis of prospectively collected data in the Swiss Eosinophilic Esophagitis Cohort Study (SEECS) and an analysis of newly collected data aimed at estimating a point prevalence of CRS in the cohort. Within the SEECS, patients undergo regular follow‐ups involving physician and patient questionnaires alongside endoscopic and histological examinations.

The SEECS represents a Swiss National Registry consisting of adult patients with a confirmed EoE diagnosis and has been established in 2015 [20, 21]. Further details on the cohort structure, recruitment process and inclusion criteria can be found in the referenced publications [20, 21]. All patients enrolled in the SEECS at the study centre of Zurich were eligible. No a priori power calculation was performed because the analysis was prevalence‐oriented and used the maximum available sample size. Recruitment was conducted from September 2025 to November 2025 via a formal invitation letter containing a link to a standardised online survey and a unique patient identifier to enable cross‐referencing with existing SEECS data.

While the SEECS provides comprehensive data on quality of life, eosinophil counts, oesophageal endoscopies and comorbid allergies, data on nasal symptoms and specifically CRS is lacking. To address this, we used a screening questionnaire comprising the four symptom‐based diagnostic criteria of the AAO‐HNS and the EPOS consensus. Both definitions vary only slightly. According to AAO‐HNS guidelines, at least two out of four symptoms must be positive for a patient to be classified with CRS, while EPOS guidelines additionally require either nasal blockage or nasal discharge to be among the positive symptoms.

The use of these four questions as a screening tool has been validated in the GA^2^LEN survey [22, 23]. In the work by Tomassen et al., 62% of subjects meeting the EPOS criteria had confirmatory endoscopic findings (adjusted OR 3.62, 95% CI 1.97–6.63) [23]. The criteria demonstrate high sensitivity (~89%) for CRS detection, as shown based on the AAO‐HNS‐definition [24], marking them suitable for ruling out CRS with reasonable confidence. Additionally, patients were inquired about a formal diagnosis of CRS by a physician, since this was shown to increase sensitivity without decreasing specificity [22, 25]. Of note, the symptom‐based diagnosis of CRS and self‐reported doctor‐diagnosed CRS were not significantly influenced by the presence of allergic rhinitis, a condition that has a symptomatic overlap with CRS and is highly prevalent in the SEECS [23].

Patients who met the CRS criteria were asked further questions, among others about CRS treatments they received and whether nasal or oesophageal symptoms affected their quality of life more strongly.

Our primary endpoint was the prevalence of CRS‐type symptoms in the cohort according to definition B, since including self‐reported doctor‐diagnosed CRS increases sensitivity as detailed above. Predefined secondary endpoints included CRS prevalence using alternative CRS definitions, alongside associations between CRS‐type symptoms and EoE patient characteristics, sinonasal and EoE symptom severities and associated comorbidities. Post hoc analyses to address potential biases included calculating CRS prevalence among patients without rhinoconjunctivitis or nasal allergies and comparing baseline characteristics, comorbidities and symptom severities of respondents with non‐respondents.

All patients who completed the online survey were included in the analysis. There was no missing data regarding questionnaire responses, as the online platform utilised mandatory response fields that prevented submission of incomplete questionnaires. Regarding SEECS, missing data were below 5% across nearly all analysed variables. The only two exceptions were peak eosinophil count at diagnosis endoscopy (68% missing) and self‐assessed symptom severity in the past 24 h at last visit (49% missing). No imputation methods were used for missing values.

All analyses were conducted using different CRS definitions, ‘A’ only including patients who reported two or more CRS symptoms in the screening questionnaire and ‘B’ including all patients from version ‘A’ plus patients who reported a pre‐existing formal diagnosis of CRS by a physician. Of note, these definitions use the AAO‐HNS guidelines. When applying EPOS guidelines, the patient number stays the same for definition B. Analyses for definition A using EPOS guidelines (definition A‐EPOS) are reported in the supplement and where differences are relevant in the main text. A fourth definition including patients with nasal polyposis according to SEECS data termed definition B‐with‐polyposis is also reported in the supplement and where relevant in the main text.

All patients provided written informed consent for participating in the SEECS; a separate informed consent form for our additional questionnaire aimed at addressing CRS was not necessary, according to inquiry at the ethical commission. Both the SEECS and this project have a valid ethical approval issued by the ethics committee of the canton of Zürich. This study is reported in accordance with the Strengthening the Reporting of Observational Studies in Epidemiology (STROBE) guidelines [26].

CRS prevalence comparisons were performed using two‐sided binomial tests. Secondary endpoints, continuous variables, including eosinophil counts and symptom scores, were analysed using the Wilcoxon rank‐sum test with continuity correction, as the data followed a non‐parametric distribution. Effect sizes were calculated using rank‐biserial tests. Categorical variables were compared using Pearson's Chi‐squared test with Yates' continuity correction and Fisher's Exact Test where frequencies were low. For calculation of odds ratios and marginal probabilities, Fisher's exact test and the R marginal effects package were used [27]. To identify and adjust for potential confounders, logistic regression models were constructed for atopic comorbidities and symptom scores. For these analyses, symptom severities were dichotomised into ‘low’ (numeric rating scale (NRS) 0–3) and ‘high’ (NRS 4–10). The same cutoff was used to categorise the severity of CRS symptom severity and was chosen in accordance with the EPOS 2020 guideline, which classifies rhinosinusitis symptom severity as mild (VAS 0–3), moderate (> 3–7) and severe (> 7–10) [28] and has been empirically validated, a score > 3.5 predicting uncontrolled CRS by EPOS criteria [29]. The cutoff is further supported by the broader NRS literature, where the mild–moderate boundary in non‐cancer chronic conditions is consistently identified at NRS 3–4 [30, 31]. In the models, we included age at first symptoms, sex, follow‐up time and atopic comorbidities as possible confounders. A *p*‐value of < 0.05 was considered statistically significant. All analyses and data visualisation were conducted using R statistical software version 4.5.2.

## 3 Results

**FIGURE 1:**
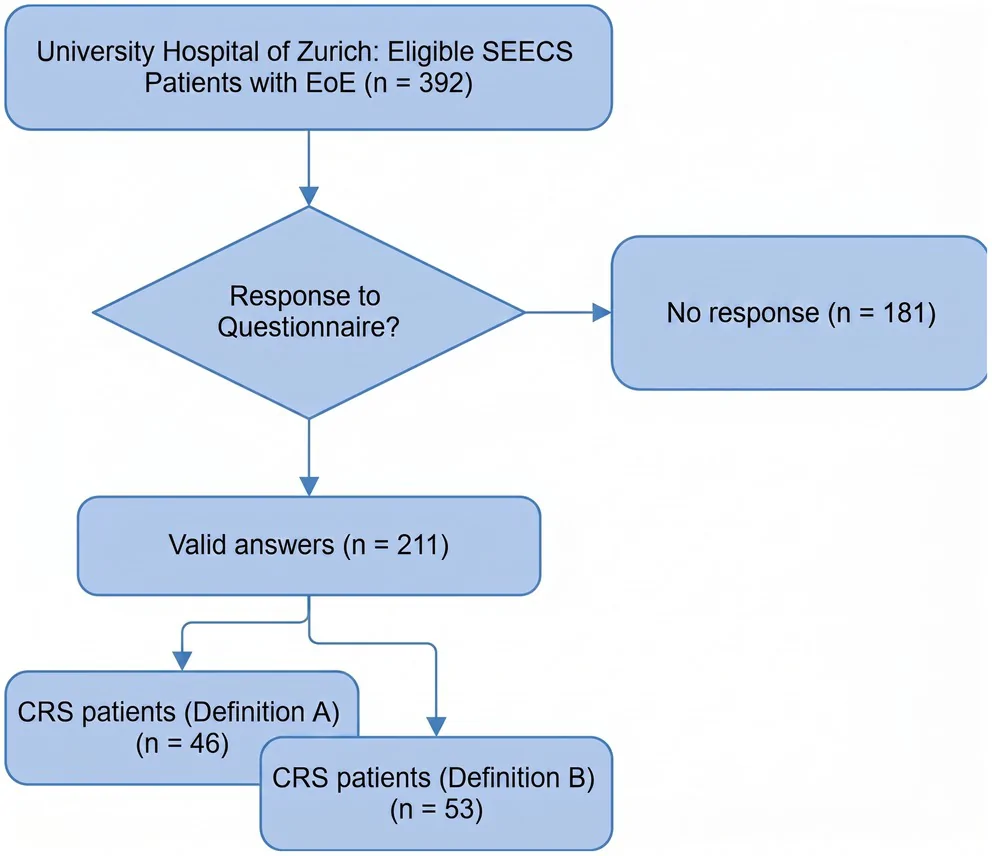
Flowchart showing the patient inclusion process.

Of the 392 SEECS patients from the Zurich study centre, 211 (53.8%) provided survey responses (Figure 1). Corresponding to the gender difference of EoE in the SEECS and other cohorts [5, 21], 161 (77.4%) of respondents were male. Only 3.8% of patients were smoking at the time of their last SEECS follow‐up visit and 24.5% had gastro‐oesophageal reflux disease (GERD) during the follow‐up period. Detailed information on baseline characteristics of our study population is provided in Table 1 (for definition A‐EPOS and B‐with‐polyposis see Table S1).

**TABLE 1:** Baseline characteristics of the study population.

	Definition A			Definition B			Overall
	CRS negative	CRS positive	*p*	CRS negative	CRS positive	*p*	All patients
*N* (%)	165 (78.2)	46 (21.8)		158 (74.9)	53 (25.1)		211 (100)
Follow‐up time, in months (mean (SD))	45.6 (32.3)	34.7 (30.5)	0.043	45.3 (32.5)	37.0 (30.9)	0.106	43.2 (32.2)
Age at first symptoms (mean (SD))	29.85 (13.74)	29.44 (14.38)	0.866	29.92 (13.69)	29.30 (14.45)	0.791	29.76 (13.85)
Age at diagnosis (mean (SD))	39.84 (12.85)	37.85 (12.16)	0.348	39.79 (13.03)	38.23 (11.68)	0.444	39.40 (12.70)
Age at last visit (mean (SD))	49.85 (13.09)	46.13 (11.98)	0.084	49.81 (13.25)	46.75 (11.70)	0.137	49.04 (12.92)
Currently smoking at last visit = Yes (%)	7 (4.3)	1 (2.2)	0.815	7 (4.5)	1 (1.9)	0.656	8 (3.8)
Ever smoked = Yes (%)	14 (8.6)	5 (10.9)	0.863	14 (9.0)	5 (9.4)	1.000	19 (9.1)
Sex = Male (%)	127 (78.4)	34 (73.9)	0.659	122 (78.7)	39 (73.6)	0.562	161 (77.4)
GERD diagnosis at screening = Yes (%)	17 (10.5)	5 (10.9)	1.000	17 (11.0)	5 (9.4)	0.956	22 (10.6)
GERD diagnosis during follow‐up = Yes (%)	40 (24.7)	11 (23.9)	1.000	39 (25.2)	12 (22.6)	0.855	51 (24.5)
Barrett's oesophagus (enrolment) = Yes (%)	3 (1.9)	0 (0.0)	0.829	3 (2.0)	0 (0.0)	0.730	3 (1.5)
Barrett's oesophagus (follow‐up) = Yes (%)	9 (6.2)	0 (0.0)	0.221	9 (6.5)	0 (0.0)	0.163	9 (4.8)

**FIGURE 2:**
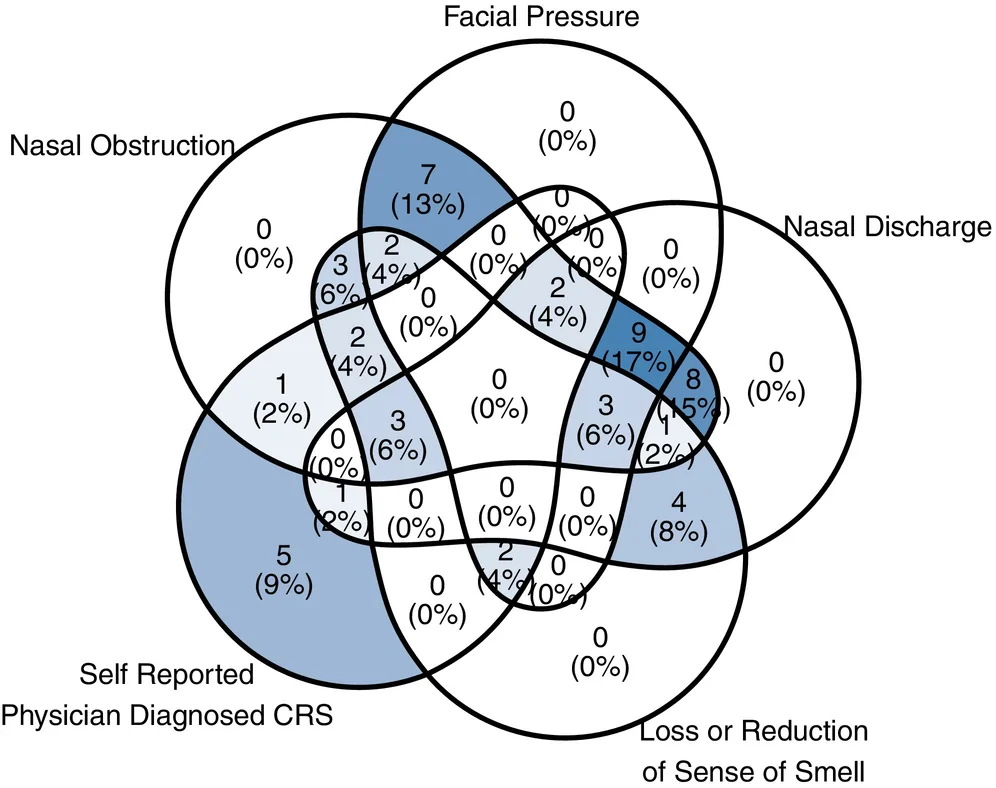
Distribution of positive screening questions and their combinations for patients meeting CRS criteria according to definition B.

Among the 53 patients with CRS‐type symptoms (definition B), nasal obstruction for at least 12 weeks was the most frequently reported symptom during the preceding 12 months (*n* = 41), followed by nasal discharge (*n* = 31) and facial pressure (*n* = 25). A loss or reduction of the sense of smell was reported by 20 patients (Figure 2). Most patients reported these symptoms to be moderate or even severe (Table 2).

**TABLE 2:** Distribution of answers to screening questions and their respective severity on a NRS in CRS positive patients (all columns except for the first based on definition B).

	All patients (*n* = 211)	CRS positive patients (*n* = 53)	Moderate (VAS 3.5–7.5)	Severe (VAS > 7.5)
12 weeks nasal obstruction	59 (28.0%)	41 (77.4%)	23/41 (56.1%)	12/41 (29.3%)
12 weeks facial pressure	29 (13.7%)	25 (47.2%)	13/25 (52.0%)	8/25 (32.0%)
12 weeks nasal discharge	33 (15.6%)	31 (58.5%)	15/31 (48.4%)	10/31 (32.3%)
12 weeks reduction sense of smell	23 (10.9%)	20 (37.7%)	11/20 (55.0%)	6/20 (30.0%)

*Note:* For symptom severities percentages refer to the number of patients who reported that symptom as positive.

Several patients reported having received CRS treatments. 49.0% (*n* = 26) said to have used topical nasal corticosteroids. 37.7% (*n* = 20) were offered a surgical treatment, of which 45.0% (*n* = 9) chose to undergo the procedure. Another 18.9% (*n* = 10) received antibiotics for CRS and 7.5% (*n* = 4) said to have been treated with Dupilumab. However, this could be imprecise since these were the same patients that received Dupilumab for their EoE.

**FIGURE 3:**
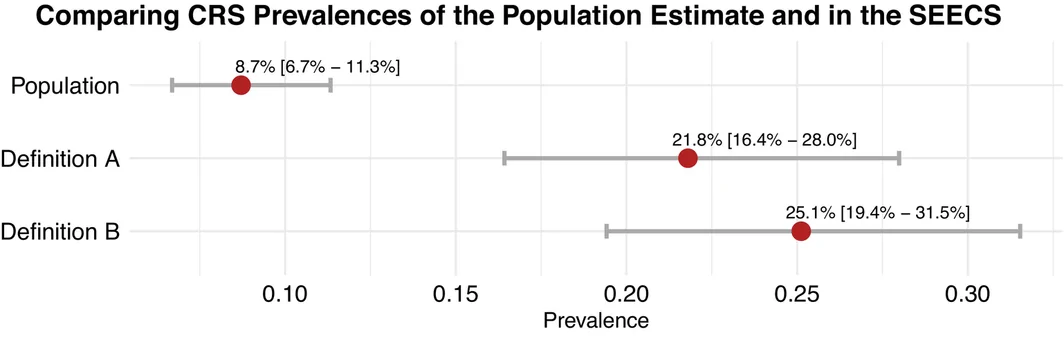
Comparing CRS point prevalence in the SEECS to the general population estimate.

Based on symptoms only (definition A), the point prevalence of CRS‐type symptoms in the cohort was 21.8% (95% CI: 16.4%–28.0%) with American Guidelines and 20.9% (95% CI: 15.6%–27.0%) with EPOS‐guidelines. According to definition B, point prevalence was 25.1% (95% CI: 19.4%–31.5%). These figures represent a significantly higher prevalence than the 8.71% prevalence observed in the general population (Figure 3). Even in comparison to the upper limit of the confidence interval for the population estimate of 11.33%, we observe a highly significant difference no matter which definition of CRS is applied (*p* < 0.001).

Since our diagnosis of CRS is symptom‐based and CRS symptoms overlap with symptoms of rhinoconjunctivitis and other nasal allergies, we also calculated the prevalence of CRS‐type symptoms in patients without these conditions. Among patients without rhinoconjunctivitis, 12.7% (definition A; 95% CI: 6.0%–22.7%) or 16.9% (definition B; 95% CI: 9.1%–27.7%) had CRS‐type symptoms (compared to 27.2% and 30.1% of rhinoconjunctivitis positive patients in definitions A and B respectively), while in patients who stated not to have nasal allergies, 12.5% (definition A; 95% CI: 5.6%–23.2%) or 15.6% (definition B; 95% CI: 7.8%–26.9%) had CRS (compared to 25.9% and 29.3% of patients with nasal allergies in definitions A and B respectively). In this scenario, the prevalence estimate of CRS‐type symptoms (definition B) in patients without rhinoconjunctivitis compared to the CRS prevalence of 8.71% stays significant (*p* = 0.03), whereas no significant difference was observed for definition A.

No significant association was observed between CRS status (with both definitions) and the oesophageal eosinophil counts (eos/hpf) at any recorded time point (enrolment, highest recorded count, most recent biopsy).

Regarding atopic comorbidities, a trend towards higher prevalence of atopic comorbidities in CRS symptom‐positive patients was observed, except for atopic dermatitis. After adjusting for sex, age at symptom onset and follow‐up duration in a multivariable regression model, this trend persisted, as can be seen in the odds ratios and marginal probabilities reported in Table 3. However, statistical significance was reached only for rhinoconjunctivitis (*p* = 0.018 in definition A; *p* = 0.0495 in definition B). The unadjusted results including definitions A‐EPOS and B‐with‐polyposis are documented in Table S2.

**TABLE 3:** Odds ratios, *p*‐values and marginal probabilities for given atopic comorbidities comparing EoE patients with and without CRS.

Comorbidity	Odds ratio CRS (Model)	*p*‐value CRS (Model)	Marginal probability CRS negative	Marginal probability CRS positive
CRS definition A				
Any atopic disease	2.31 (0.88–7.33)	0.1159	76.8% (69.9–83.7)	88.2% (84.0–92.3)
Oral allergy syndrome	1.34 (0.65–2.76)	0.4284	40.7% (32.7–48.6)	47.5% (39.2–55.7)
Asthma	1.08 (0.5–2.26)	0.8333	32.4% (24.6–40.1)	34.1% (26.2–42.1)
Rhinoconjunctivitis	3.08 (1.3–8.22)*	0.0154	62.0% (54.1–69.9)	82.7% (77.8–87.7)
Atopic dermatitis	0.14 (0.01–0.7)	0.0574	14.5% (8.7–20.3)	2.3% (1.2–3.4)
CRS definition B				
Any atopic disease	1.74 (0.72–4.69)	0.2417	77.3% (70.3–84.3)	85.3% (80.3–90.4)
Oral allergy syndrome	1.38 (0.69–2.75)	0.3609	40.2% (32.1–48.4)	47.7% (39.3–56.2)
Asthma	0.94 (0.45–1.91)	0.8742	33.1% (25.1–41.1)	31.8% (24.0–39.7)
Rhinoconjunctivitis	2.24 (1.03–5.23)*	0.0495	62.6% (54.6–70.6)	78.4% (72.4–84.3)
Atopic dermatitis	0.25 (0.04–0.92)	0.071	14.4% (8.5–20.3)	4.1% (2.2–6.1)

*p < 0.05.

In terms of disease perception, many CRS‐type‐symptom‐positive patients (i.e., *n* = 17, 32%) indicated that both EoE and CRS affected their quality of life only mildly. However, a notable subset reported a substantial burden: 25% (*n* = 13) perceived their CRS symptoms as more debilitating than their EoE symptoms, while 13% (*n* = 7) reported that both conditions severely impacted their quality of life. The remaining patients reported that their EoE symptoms impacted their life more strongly than their CRS symptoms.

**FIGURE 4:**
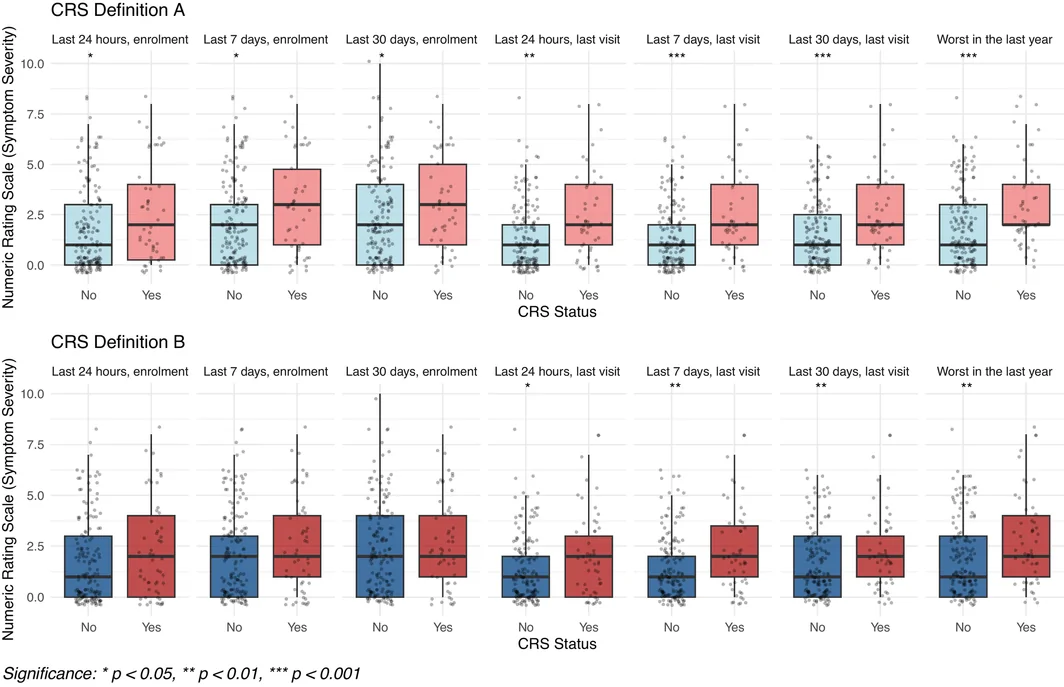
Boxplots showing symptom severity at different time points and across both definitions of CRS in patients with concomitant CRS and patients without.

This was also reflected in physician‐reported EoE symptom severity. Patients with concomitant CRS‐type symptoms (definition A) exhibited significantly higher EoE symptom severity scores across all observed time points and durations (at enrolment and last visit symptom burden in the past 24 h, 7 and 30 days). Under definition B, this difference remained significant only at the last follow‐up, but trends stayed the same (Figure 4) (for odds ratios, *p*‐values and effect sizes as well as definition A‐EPOS and B‐with‐polyposis see Table S3). As shown in Table 4, in the generalised logistic models, EoE symptom severity at last visit remained a significant predictor of CRS status. However, shorter follow‐up duration and, to a lesser extent, the presence of atopic comorbidities were identified as significant confounding factors influencing this association (Table 4, for definitions A‐EPOS and B‐with‐polyposis see Table S4). Nevertheless, odds ratios and marginal probabilities, as can be seen in Figure S1 and Table 4 show a clear trend towards higher symptom severities in all models.

**TABLE 4:** Odds ratios and marginal probabilities for generalised linear models comparing the risk for high symptom severity (NRS > 3) including odds ratios for possible confounders included in the models.

	Past 24 h (enrolment)	Past 7 days (enrolment)	Past 30 days (enrolment)	Past 24 h (last visit)	Past 7 days (last visit)	Past 30 days (last visit)	Worst (all visits)
CRS definition A							
Intercept	0.24 (0.04–1.09)	0.17 (0.03–0.73)*	0.41 (0.1–1.59)	1.29 (0.26–6.12)	1.62 (0.34–7.66)	1.21 (0.25–5.58)	3.25 (0.79–13.9)
CRS	1.64 (0.71–3.69)	1.93 (0.89–4.13)	1.84 (0.86–3.89)	2.99 (1.17–7.58)*	3.55 (1.4–9.06)**	2.85 (1.13–7.13)*	2.53 (1.08–5.91)*
Age at first symptoms	1 (0.97–1.02)	0.99 (0.97–1.02)	0.98 (0.96–1.01)	0.97 (0.94–1)	0.97 (0.94–1)	0.98 (0.94–1.01)	0.97 (0.94–0.99)*
Sex (male)	1.04 (0.43–2.72)	1.19 (0.51–2.94)	1.02 (0.46–2.38)	0.57 (0.22–1.58)	0.47 (0.18–1.28)	0.61 (0.24–1.66)	0.6 (0.25–1.5)
Any atopic disease	2.84 (0.98–10.43)	3.33 (1.19–11.89)*	2.25 (0.9–6.47)	0.51 (0.18–1.51)	0.41 (0.15–1.19)	0.43 (0.16–1.22)	0.41 (0.16–1.03)
Follow‐up time, in months	0.98 (0.97–0.99)**	0.99 (0.98–1)	0.99 (0.98–1)	0.99 (0.97–1)	0.98 (0.97–1)*	0.99 (0.97–1)	0.98 (0.97–1)**
Marginal Prob: CRS negative	19.6% (3.6%–35.6%)	23% (4.9%–41.1%)	25.9% (9.2%–42.5%)	11.1% (2.6%–19.6%)	11% (2.9%–19.2%)	11.8% (3.3%–20.2%)	17.6% (7.2%–28%)
Marginal Prob: CRS positive	27.8% (7.9%–47.8%)	35.8% (12.7%–58.9%)	38.4% (18.1%–58.7%)	25.6% (9.8%–41.4%)	28.2% (12.3%–44.1%)	26.2% (10.9%–41.4%)	32.7% (17.6%–47.8%)
CRS definition B							
Intercept	0.25 (0.05–1.12)	0.18 (0.03–0.75)*	0.43 (0.1–1.64)	1.29 (0.26–6.09)	1.6 (0.34–7.51)	1.22 (0.26–5.6)	3.16 (0.76–13.53)
CRS	1.31 (0.57–2.9)	1.5 (0.7–3.15)	1.41 (0.67–2.91)	2.32 (0.93–5.71)	2.7 (1.1–6.67)*	2.18 (0.88–5.3)	2.23 (0.98–5.03)
Age at first symptoms	1 (0.97–1.02)	0.99 (0.97–1.02)	0.99 (0.96–1.01)	0.97 (0.94–1)	0.97 (0.94–1.01)	0.98 (0.94–1.01)	0.97 (0.94–0.99)*
Sex (male)	1.04 (0.43–2.71)	1.18 (0.51–2.92)	1.02 (0.46–2.36)	0.56 (0.22–1.54)	0.46 (0.18–1.24)	0.59 (0.23–1.62)	0.59 (0.25–1.46)
Any atopic disease	2.93 (1.02–10.75)	3.46 (1.25–12.32)*	2.34 (0.94–6.71)	0.55 (0.2–1.61)	0.46 (0.17–1.29)	0.47 (0.18–1.3)	0.43 (0.17–1.08)
Follow‐up time, in months	0.98 (0.97–0.99)**	0.99 (0.98–1)	0.99 (0.98–1)	0.98 (0.97–1)*	0.98 (0.97–1)*	0.99 (0.97–1)	0.98 (0.97–0.99)**
Marginal Prob: CRS negative	20.4% (4.1%–36.8%)	24% (5.4%–42.6%)	27% (9.9%–44.1%)	11.6% (2.7%–20.6%)	11.6% (3.1%–20.1%)	12.3% (3.4%–21.3%)	17.7% (7.1%–28.3%)
Marginal Prob: CRS positive	24.8% (6.2%–43.4%)	31.7% (9.9%–53.6%)	33.9% (14.6%–53.2%)	22.3% (7.8%–36.9%)	24.5% (9.8%–39.3%)	22.7% (8.6%–36.7%)	30.5% (15.7%–45.3%)

*p < 0.05; **p < 0.01.

We further evaluated the impact of comorbid CRS‐type symptoms on longitudinal EoE symptom severity. Comparisons were made between scores at enrolment and the most recent follow‐up visit. Direct comparisons of symptom improvement (Δ NRS) did not reach statistical significance, but a diverging trend was observed. Patients without comorbid CRS exhibited significant improvement in symptom severity over the 7‐ and 30‐day recall periods, while patients with CRS symptoms showed no statistically significant improvement across any timeframe. However, testing for confounders revealed that this effect was mainly due to a shorter follow‐up period.

CRS status was not associated with the presence of EoE symptoms such as thoracic retrosternal pain, odynophagia, heartburn, volume regurgitation, vomiting or abdominal pain at baseline and at last visit. Only dysphagia for solids at last visit was significantly associated with CRS definition A, but not with CRS definition B.

To address a potential response bias, we analysed baseline characteristics, comorbidities and symptom severities of responders and non‐responders (for details, see Table S5). In univariate analyses, responders had a significantly longer mean follow‐up period compared to non‐responders (42.97 vs. 35.60 months, *p* = 0.025), as well as a higher mean age at their last clinical visit (49.04 vs. 44.97 years, *p* = 0.003). No significant differences were observed between responders and non‐responders regarding sex, atopic comorbidities, GERD, Barrett's oesophagus or EoE symptom severity scores. Interestingly, at the last visit for both the past 7 days and the past 30 days a trend towards higher symptom severities among non‐respondents was observed. This was consistent with results from a multivariable logistic regression model predicting response status, where age at diagnosis (OR = 1.016, 95% CI: 1.001–1.031) and total follow‐up time in months (OR = 1.007, 95% CI: 1.002–1.014) remained a significant independent predictor of questionnaire completion.

## 4 Discussion

To the best of our knowledge, this is the first study to show that CRS‐type symptoms are a highly frequent and most likely under‐recognised comorbidity in EoE patients with a striking prevalence of around one in four EoE patients affected—a figure two to three times higher compared to the general population. These results align with existing data regarding the increased prevalence of concomitant atopic diseases in EoE patients, as well as the elevated EoE prevalence observed in CRS patients.

Several key findings emerge from our analysis. First, the point prevalence of CRS‐type symptoms in our EoE cohort was 20.9% (definition A‐EPOS) to 25.1% (definition B), representing a striking increase compared to the general population estimate of 8.71%. We consider definition B the best suited to assess CRS prevalence, because including self‐reported doctor‐diagnosed CRS increases sensitivity without negatively affecting specificity [22] and patients reporting doctor‐diagnosed CRS also stated to have received CRS‐specific treatments. Second, patients with CRS‐type symptoms exhibited significantly higher EoE symptom severity consistently under definition A and, under the primary definition B, at the last visit; this association persisted after adjustment for confounders under definition A. Third, a quarter of patients with concomitant CRS‐type symptoms perceived their nasal symptoms as more debilitating than their EoE and quantified symptom severity assessment revealed that most CRS‐symptom‐positive patients reported at least moderate‐to‐severe nasal symptoms according to the EPOS 2020 classification. These findings substantially expand the limited existing data on the EoE–CRS association, which until now has been examined almost exclusively from the otorhinolaryngological perspective, documenting increased EoE prevalence in CRS patients but not the reverse.

Despite the observed clinical overlap, no significant association between oesophageal eosinophil counts and CRS status was found. This suggests that while both diseases may share an underlying Th2‐inflammatory predisposition, the local tissue manifestation of eosinophilia in the oesophagus does not necessarily mirror sinonasal inflammation. Furthermore, after adjusting for confounders, EoE symptom severity improvements over time and individual EoE symptom frequencies did not differ between the CRS‐positive and CRS‐negative groups. While this may suggest that CRS status does not strongly impact histological disease activity of EoE or EoE treatment response, these results should be interpreted cautiously, as this study was not designed to compare treatment response.

Another finding is that patients with both diseases also had a trend towards higher probabilities of other atopic comorbidities, an effect that only reached statistical significance in rhinoconjunctivitis. However, this is not surprising, since this condition has a relevant symptom overlap with CRS. There was no correlation between CRS status and GERD in our EoE patients.

An intriguing aspect relates to the well‐established association between CRS and GERD. Given that EoE is frequently misdiagnosed as GERD [32, 33], particularly outside gastroenterology and that the median diagnostic delay for EoE remains 6 years [34], it is conceivable that a proportion of patients classified as having CRS with GERD may harbour undiagnosed EoE—a hypothesis that warrants investigation.

Our observation that CRS‐type‐symptom‐positive EoE patients reported consistently higher symptom severity and that a substantial subset perceived their CRS‐type symptoms as more burdensome than their primary oesophageal disease, has important clinical implications. It is plausible that a comparable phenomenon exists in reverse: some CRS patients under otorhinolaryngological care may have unrecognised EoE contributing significantly to their overall symptom burden. This bidirectional pattern is not unique to the EoE–CRS dyad but likely extends to other type 2 inflammatory conditions. Patients with severe asthma, for instance, frequently report impaired quality of life driven not by airway obstruction alone but by comorbid CRS, atopic dermatitis or eosinophilic gastrointestinal disease and there is growing evidence that addressing these comorbidities improves overall disease control [35, 36]. Our findings add further evidence to the emerging paradigm that type 2 inflammatory diseases should not be viewed in isolation but as interconnected manifestations of a shared immunological predisposition whose cumulative impact on patients exceeds the sum of its parts.

Importantly, not all patients with atopic comorbidities will require systemic therapy targeting the shared type 2 inflammatory pathway. Topical corticosteroids, dietary interventions and organ‐specific approaches remain the cornerstone of management for the majority. However, for selected patients with a substantial combined symptom burden across multiple organs, biologic agents such as Dupilumab offer the unique opportunity to address multiple disease manifestations with a single treatment [16, 17, 37]. Critically, however, such treatment decisions can only be made if clinicians are aware of the full spectrum of their patients' type 2 comorbidities. Our results, together with recent initiatives such as the Delphi‐validated type 2 inflammation screening questionnaire [38, 39] support the imperative for systematic cross‐speciality screening. Gastroenterologists should actively inquire about nasal and respiratory symptoms in EoE patients, just as otorhinolaryngologists should consider oesophageal involvement in CRS patients. Only through such interdisciplinary awareness can clinicians identify those patients who stand to benefit most from integrated, pathway‐directed treatment, thereby reducing overall disease burden and improving quality of life.

While this study benefits from the use of a well‐characterised EoE cohort, several limitations must be acknowledged. Firstly, the diagnosis of CRS was questionnaire‐based. The use of the questionnaire has been validated as a screening tool; however, formal diagnosis requires CT imaging or endoscopy. Therefore, our definition of CRS does not equal an ENT specialist diagnosis of CRS. This is also the reason why we rather use the term CRS‐type symptoms than CRS in this paper. Secondly, the absence of an enrolled control group necessitates comparison against general population prevalence data, which may increase the risk of bias. There is no robust prevalence data in Switzerland on a population basis for CRS (and for EoE), which results in comparing the prevalence in our Swiss cohort with an international, geographically different and diverse population prevalence. Thirdly, the 53.8% response rate raises the possibility of responder bias, which risks overestimating disease prevalence since symptomatic patients might be inclined to respond preferentially. To address this, we compared characteristics of responders and non‐responders. In a multivariable analysis, a shorter follow‐up correlated with a lower response probability. Interestingly, among respondents, patients with shorter follow‐up periods were those more likely to have CRS‐like symptoms and had a trend towards greater EoE symptom severity scores. Therefore, while the low response rate raises concerns about a responder bias overestimating CRS prevalence, we found no evidence that responders were preferentially more symptomatic, though residual bias cannot be excluded.

The symptom overlap between CRS‐type symptoms and allergic rhinitis, which is highly prevalent in EoE, could in principle inflate our prevalence estimate and our symptom‐based figures should be interpreted with this caveat in mind. However, several observations argue that overlap alone does not explain our findings. Even among EoE patients without rhinoconjunctivitis, CRS‐type symptoms remained nearly twice as prevalent as in the general population (16.9% vs. 8.71% under our primary endpoint, definition B; *p* = 0.03). Moreover, that CRS‐type symptoms cluster with rhinoconjunctivitis (30.1% vs. 16.9% in definition B) is itself consistent with our central hypothesis of a shared type 2 inflammatory diathesis, in which their co‐occurrence is expected rather than confounding. Therefore, the rhinoconjunctivitis‐negative estimate (16.9%) provides, if anything, a conservative lower bound, more likely under‐stating than over‐stating the recognised burden of CRS in EoE. Consistent with genuine sinonasal disease rather than misattributed rhinitis, Tomassen et al. showed that symptom‐based CRS predicts positive nasal endoscopy irrespective of allergic rhinitis status [23].

Neither age nor sex was adequately matched; in particular, age‐stratified estimates from some general‐population studies report CRS prevalence up to 12%–15% in adults of comparable age range, which would attenuate the magnitude—but not the statistical significance—of the difference observed in our cohort. The robustness of this finding is further supported by the consistency of results across the multiple CRS definitions applied. Given the exploratory nature of our secondary endpoint analyses and the inherent correlation between the outcomes, no formal correction for multiple testing was used. Nevertheless, we considered our findings robust based on the consistency of results across related clinical endpoints.

In conclusion, CRS‐type symptoms are a frequent and impactful comorbidity in patients with EoE. The presence of CRS‐type symptoms is associated with a higher perceived EoE symptom burden. This highlights the importance of an increased awareness among specialists treating type 2 diseases for potential comorbid conditions, an intensification of interdisciplinary exchange to better understand, as well as more effectively diagnose and address these comorbidities and further research on the topic.

## Author Contributions

**Andrea Kreienbühl:** writing – review and editing, conceptualization, methodology, writing – original draft. **Thomas Greuter:** writing – review and editing, writing – original draft. **Aidan Gebhardt:** conceptualization, writing – original draft, writing – review and editing, formal analysis, investigation, visualization, methodology. **Jean‐Benoit Rossel:** formal analysis, writing – review and editing, writing – original draft, resources. **Alain Schoepfer:** funding acquisition, writing – review and editing, writing – original draft. **Catherine Saner:** conceptualization, methodology, writing – original draft, resources. **Christoph Schlag:** writing – review and editing, writing – original draft. **Michael B. Soyka:** writing – review and editing, conceptualization, methodology, writing – original draft. **Luc Biedermann:** supervision, conceptualization, methodology, writing – review and editing, investigation, formal analysis, validation, visualization, project administration, writing – original draft. **Alex Straumann:** writing – review and editing, conceptualization, methodology, writing – original draft. **Christian M. Meerwein:** writing – review and editing, writing – original draft. **Simon Nennstiel:** writing – review and editing, writing – original draft. **Tina Mauthe:** writing – review and editing, writing – original draft.

## Funding

This work was supported by Schweizerischer Nationalfonds zur Förderung der Wissenschaftlichen Forschung (32003B_204751/1) and Swiss EoE Foundation.

## Ethics Statement

The study protocol of the SEECS including all related questionnaires was reviewed and approved by the Local Ethics Committees at each of the participating sites (lead commission CER‐VD, Approval No. 148‐15). A protocol amendment was approved by CER‐VD in July 2022 after REDCapping of questionnaires (PB_2016_01962 [148–15]). Patients provided their written informed consent to participate in this study. The study protocol of our project including the questionnaire was approved by the Local Ethics Committee of the Canton of Zurich (No. 2024‐01979).

## Supporting information


**Table S1:** Baseline characteristics of the study population: two alternative CRS definitions.
**Table S2:** Odds ratios calculated with Fisher's exact test for CRS and atopic comorbidities using all four CRS definitions.
**Table S3:** Odds ratios, *p*‐values and effect sizes across different CRS‐definitions and timeframes.
**Table S4:** Odds ratios and marginal probabilities for generalised linear models comparing the risk for high symptom severity (NRS > 3) including odds ratios for possible confounders included in the models in the additional CRS definitions.
**Table S5:** Comparison of baseline characteristics, EoE symptom severities and comorbidities of responders and non‐responders to the additional CRS questionnaire.

**Figure S1:** Marginal probabilities of high symptom severity stratified by CRS status at different time points. Asterisks' indicate statistically significant differences (* for *p* < 0.05, ** for *p* < 0.01).

## Data Availability

The data that support the findings of this study are not publicly available as patients enrolled into SEECS did not consent to make their data publicly available. The new data was collected in the framework of the SEECS, and the patients did not sign a separate informed consent form, thus this also applies to the new data. Requests for access to SEECS data in the framework of a clearly defined research project can be submitted to the scientific board of the SEECS.
