# Supplementary material for: Eosinophilic Oesophagitis Beyond the Oesophagus: High Prevalence of Chronic Rhinosinusitis‐Type Symptoms and Their Impact on Symptom Severity in Swiss EoE Cohort Study Patients
Source: Aliment Pharmacol Ther. 2026 Jun 25;64(7):965–75. doi: 10.1111/apt.70819 (PMC13559487; doi:10.1111/apt.70819)
Supplement: Supplementary file 1 [file APT-64-965-s002.docx]

**Supplement**

|  | Definition A-EPOS | | | Definition B-with-polyposis | | |
| --- | --- | --- | --- | --- | --- | --- |
|  | **CRS negative** | **CRS positive** | **p-value** | **CRS negative** | **CRS positive** | **p-value** |
| n | 167 (79.1%) | 44 (20.9%) |  | 155 (73.5%) | 56 (26.5%) |  |
| Follow-up time, in months (mean (SD)) | 45.18 (32.40) | 35.84 (30.69) | 0.087 | 46.04 (32.33) | 35.52 (30.81) | 0.036 |
| Age at first symptoms (mean (SD)) | 30.05 (13.75) | 28.69 (14.33) | 0.589 | 29.87 (13.72) | 29.46 (14.33) | 0.861 |
| Age at diagnosis (mean (SD) | 39.94 (12.81) | 37.41 (12.23) | 0.242 | 39.61 (13.07) | 38.82 (11.70) | 0.692 |
| Age at last visit (mean (SD)) | 49.84 (13.02) | 46.00 (12.22) | 0.079 | 49.76 (13.33) | 47.05 (11.58) | 0.180 |
| Currently smoking at last visit = Yes (%) | 7 (4.3) | 1 (2.3) | 0.865 | 7 (4.6) | 1 (1.8) | 0.595 |
| Ever smoked = Yes (%) | 14 (8.5) | 5 (11.4) | 0.777 | 14 (9.2) | 5 (8.9) | 1 |
| Sex = Male (%) | 129 (78.7) | 32 (72.7) | 0.527 | 120 (78.9) | 41 (73.2) | 0.49 |
| GERD diagnosis at screening = Yes (%) | 18 (11.0) | 4 (9.1) | 0.932 | 16 (10.5) | 6 (10.7) | 1 |
| GERD diagnosis during follow-up = Yes (%) | 41 (25.0) | 10 (22.7) | 0.909 | 38 (25.0) | 13 (23.2) | 0.933 |
| Barrett's oesophagus (enrolment) = Yes (%) | 3 (1.9) | 0 (0.0) | 0.859 | 3 (2.0) | 0 (0.0) | 0.691 |
| Barrett's oesophagus (follow-up) = Yes (%) | 9 (6.1) | 0 (0.0) | 0.244 | 9 (6.6) | 0 (0.0) | 0.147 |

Supplementary Table S1 Baseline characteristics of the study population: two alternative CRS definitions

|  | Probability CRS positive (95% CI) | Probability CRS negative (95% CI) | Odds Ratio and 95% CI | p_value |
| --- | --- | --- | --- | --- |
| **CRS Definition A** | | | | |
| Any atopic disease | 89.1% (76.4-96.4) | 77.6% (70.4-83.8) | 2.35 (0.84-8.19) | 0.0961 |
| Oral allergy syndrome | 47.8% (32.9-63.1) | 39.8% (32.1-47.8) | 1.39 (0.68-2.83) | 0.3967 |
| Asthma | 37.0% (23.2-52.5) | 32.9% (25.7-40.8) | 1.19 (0.56-2.48) | 0.6014 |
| Rhinoconjunctivitis | 80.4% (66.1-90.6) | 61.5% (53.5-69.0) | 2.56 (1.12-6.47) | 0.0214 |
| Neurodermitis | 6.5% (1.4-17.9) | 15.5% (10.3-22.1) | 0.38 (0.07-1.34) | 0.145 |
| **CRS Definition B** | | | | |
| Any atopic disease | 86.8% (74.7-94.5) | 77.9% (70.5-84.2) | 1.86 (0.74-5.32) | 0.2299 |
| Oral allergy syndrome | 49.1% (35.1-63.2) | 39.0% (31.2-47.1) | 1.51 (0.76-2.97) | 0.2579 |
| Asthma | 35.8% (23.1-50.2) | 33.1% (25.8-41.1) | 1.13 (0.55-2.27) | 0.7383 |
| Rhinoconjunctivitis | 77.4% (63.8-87.7) | 61.7% (53.5-69.4) | 2.11 (0.99-4.79) | 0.0444 |
| Neurodermitis | 9.4% (3.1-20.7) | 14.9% (9.7-21.6) | 0.59 (0.17-1.72) | 0.3617 |
| **CRS Definition A-EPOS** | | | | |
| Any atopic disease | 88.6% (75.4-96.2) | 77.9% (70.8-84.0) | 2.20 (0.78-7.69) | 0.1377 |
| Oral allergy syndrome | 47.7% (32.5-63.3) | 39.9% (32.3-47.8) | 1.37 (0.66-2.84) | 0.3906 |
| Asthma | 34.1% (20.5-49.9) | 33.7% (26.5-41.6) | 1.02 (0.47-2.15) | 1 |
| Rhinoconjunctivitis | 79.5% (64.7-90.2) | 62.0% (54.0-69.4) | 2.38 (1.03-6.02) | 0.0322 |
| Neurodermitis | 4.5% (0.6-15.5) | 16.0% (10.7-22.5) | 0.25 (0.03-1.08) | 0.0501 |
| **CRS Definition B-with-polyposis** | | | | |
| Any atopic disease | 87.5% (75.9-94.8) | 77.5% (70.0-83.9) | 2.03 (0.81-5.79) | 0.1203 |
| Oral allergy syndrome | 46.4% (33.0-60.3) | 39.7% (31.9-48.0) | 1.31 (0.67-2.55) | 0.4288 |
| Asthma | 35.7% (23.4-49.6) | 33.1% (25.7-41.2) | 1.12 (0.56-2.23) | 0.7428 |
| Rhinoconjunctivitis | 76.8% (63.6-87.0) | 61.6% (53.3-69.4) | 2.06 (0.98-4.54) | 0.0481 |
| Neurodermitis | 8.9% (3.0-19.6) | 15.2% (9.9-22.0) | 0.55 (0.15-1.58) | 0.3596 |

Supplementary Table S2 Odds ratios calculated with Fisher’s exact test for CRS and atopic comorbidities using all four CRS definitions

| SymptomSeverity | Past 24 hours at enrolment | Past 7 days at enrolment | Past 30 days at enrolment | Past 24 hours at last visit | Past 7 days at last visit | Past 30 days at last visit | Worst in the last year |
| --- | --- | --- | --- | --- | --- | --- | --- |
| CRS Definition A | | | | | | | |
| Odds Ratio (95% CI) | 1.05 (1 - 1.19) | 1.04 (1 - 1.16) | 1.04 (1 - 1.16) | 1.1 (1.01 - 1.27) | 1.13 (1.03 - 1.33) | 1.11 (1.02 - 1.29) | 1.13 (1.03 - 1.33) |
| Effect Size | 0.226 | 0.203 | 0.2 | 0.299 | 0.338 | 0.315 | 0.344 |
| P-Value | 0.0151 | 0.0323 | 0.0358 | 0.0015 | 4.00E-04 | 0.001 | 3.00E-04 |
| CRS Definition B | | | | | | | |
| Odds Ratio (95% CI) | 1.03 (1 - 1.13) | 1.02 (1 - 1.1) | 1.02 (1 - 1.1) | 1.05 (1 - 1.18) | 1.07 (1.01 - 1.21) | 1.06 (1 - 1.19) | 1.09 (1.01 - 1.24) |
| Effect Size | 0.169 | 0.13 | 0.132 | 0.224 | 0.253 | 0.238 | 0.282 |
| P-Value | 0.0565 | 0.1495 | 0.1456 | 0.013 | 0.0055 | 0.0093 | 0.002 |
| CRS Definition A-EPOS | | | | | | | |
| Odds Ratio (95% CI) | 1.06 (1 - 1.2) | 1.05 (1 - 1.18) | 1.05 (1 - 1.18) | 1.1 (1.01 - 1.28) | 1.14 (1.03 - 1.34) | 1.1 (1.01 - 1.27) | 1.13 (1.02 - 1.32) |
| Effect Size | 0.233 | 0.214 | 0.213 | 0.304 | 0.347 | 0.296 | 0.334 |
| P-Value | 0.014 | 0.0266 | 0.0276 | 0.0015 | 3.00E-04 | 0.0023 | 6.00E-04 |
| CRS-Definition B-with-polyposis | | | | | | | |
| Odds Ratio (95% CI) | 1.04 (1 - 1.16) | 1.03 (1 - 1.12) | 1.03 (1 - 1.12) | 1.06 (1.01 - 1.2) | 1.08 (1.01 - 1.22) | 1.07 (1.01 - 1.2) | 1.09 (1.01 - 1.24) |
| Effect Size | 0.206 | 0.16 | 0.159 | 0.246 | 0.271 | 0.253 | 0.286 |
| P-Value | 0.0183 | 0.0704 | 0.0752 | 0.0055 | 0.0024 | 0.0049 | 0.0014 |

Supplementary Table S3 Odds ratios, p-values and effect sizes across different CRS-definitions and timeframes

|  | Past 24 hours (enrolment) | Past 7 days (enrolment) | Past 30 days (enrolment) | Past 24 hours (last visit) | Past 7 days (last visit) | Past 30 days (last visit) | Worst (all visits) |
| --- | --- | --- | --- | --- | --- | --- | --- |
| **CRS definition A-EPOS** | | | | | | | |
| Intercept | 0.23 (0.04-1.08) | 0.17 (0.03-0.72)* | 0.41 (0.1-1.57) | 1.28 (0.26-6.07) | 1.58 (0.33-7.49) | 1.21 (0.25-5.56) | 3.19 (0.77-13.64) |
| CRS | 1.58 (0.67-3.6) | 1.87 (0.85-4.05) | 1.78 (0.82-3.81) | 2.56 (0.99-6.46)* | 3.04 (1.19-7.7)* | 2.45 (0.95-6.13) | 2.27 (0.96-5.32) |
| Age at first symptoms | 1 (0.97-1.02) | 0.99 (0.97-1.02) | 0.99 (0.96-1.01) | 0.97 (0.94-1) | 0.97 (0.94-1.01) | 0.98 (0.94-1.01) | 0.97 (0.94-0.99)* |
| Sex (Male) | 1.05 (0.43-2.74) | 1.2 (0.52-2.97) | 1.03 (0.46-2.4) | 0.58 (0.23-1.6) | 0.49 (0.19-1.3) | 0.62 (0.24-1.68) | 0.61 (0.26-1.52) |
| Any atopic disease | 2.89 (1-10.63) | 3.39 (1.22-12.12)* | 2.29 (0.92-6.58) | 0.53 (0.19-1.58) | 0.44 (0.16-1.25) | 0.46 (0.17-1.27) | 0.42 (0.17-1.07) |
| Follow-up time, in months | 0.98 (0.97-0.99)** | 0.99 (0.98-1) | 0.99 (0.98-1) | 0.98 (0.97-1)* | 0.98 (0.97-1)* | 0.99 (0.97-1) | 0.98 (0.97-0.99)** |
| Marginal Prob: CRS negative | 19.9% (3.8-36%) | 23.3% (5-41.6%) | 26.2% (9.4-43%) | 11.7% (2.8-20.6%) | 11.7% (3.2-20.2%) | 12.4% (3.5-21.2%) | 18.2% (7.5-28.8%) |
| Marginal Prob: CRS positive | 27.5% (7.6-47.3%) | 35.5% (12.5-58.5%) | 38.1% (17.9-58.3%) | 24.1% (9-39.3%) | 26.7% (11.3-42.2%) | 24.6% (10-39.3%) | 31.5% (16.7-46.3%) |
| **CRS definition B-with-polyposis** | | | | | | | |
| Intercept | 0.24 (0.04-1.1) | 0.17 (0.03-0.74)* | 0.42 (0.1-1.62) | 1.25 (0.25-5.95) | 1.56 (0.33-7.4) | 1.18 (0.25-5.45) | 3.13 (0.75-13.43) |
| CRS | 1.45 (0.64-3.18) | 1.64 (0.77-3.43) | 1.53 (0.73-3.14) | 2.76 (1.11-6.82)* | 3.21 (1.31-8)* | 2.58 (1.06-6.29)* | 2.53 (1.12-5.73)* |
| Age at first symptoms | 0.99 (0.97-1.02) | 0.99 (0.97-1.02) | 0.98 (0.96-1.01) | 0.97 (0.94-1) | 0.97 (0.94-1) | 0.98 (0.94-1.01) | 0.97 (0.94-0.99)* |
| Sex (Male) | 1.03 (0.43-2.7) | 1.18 (0.51-2.91) | 1.01 (0.46-2.36) | 0.55 (0.21-1.51) | 0.45 (0.17-1.21) | 0.58 (0.23-1.59) | 0.58 (0.24-1.44) |
| Any atopic disease | 2.88 (1-10.57) | 3.42 (1.23-12.17)* | 2.32 (0.93-6.64) | 0.53 (0.19-1.57) | 0.44 (0.16-1.24) | 0.45 (0.17-1.27) | 0.42 (0.17-1.06) |
| Follow-up time, in months | 0.98 (0.97-0.99)** | 0.99 (0.98-1) | 0.99 (0.98-1) | 0.99 (0.97-1)* | 0.98 (0.97-1)* | 0.99 (0.97-1) | 0.98 (0.97-1)** |
| Marginal Prob: CRS negative | 19.9% (3.8-36.1%) | 23.5% (5.1-41.8%) | 26.5% (9.6-43.4%) | 10.9% (2.4-19.5%) | 10.9% (2.7-19%) | 11.6% (3.1-20.2%) | 17.1% (6.7-27.4%) |
| Marginal Prob: CRS positive | 25.9% (6.8-45%) | 32.9% (10.6-55.2%) | 35.1% (15.5-54.7%) | 23.9% (8.6-39.3%) | 26.1% (10.6-41.5%) | 24.3% (9.5-39.1%) | 31.9% (16.7-47.1%) |

Supplementary Table S4 Odds ratios and marginal probabilities for generalised linear models comparing the risk for high symptom severity (NRS > 3) including odds ratios for possible confounders included in the models in the additional CRS definitions.

|  | 0 | 1 | p-value |
| --- | --- | --- | --- |
| n (%) | 181 (46.2) | 211 (53.8) |  |
| Follow-up time, in months (mean (SD)) | 35.60 (32.50) | 42.97 (32.23) | 0.025* |
| Age at first symptoms (mean (SD)) | 29.53 (14.47) | 29.76 (13.85) | 0.881 |
| Age at diagnosis (mean (SD)) | 36.76 (14.03) | 39.40 (12.70) | 0.053 |
| Age at last visit (mean (SD)) | 44.97 (13.79) | 49.04 (12.92) | 0.003* |
| Sex = Male (%) | 132 (72.9) | 163 (77.3) | 0.384 |
| GERD diagnosis at enrolment = Yes (%) | 36 (20.3) | 29 (13.9) | 0.12 |
| GERD diagnosis during follow-up = Yes (%) | 43 (23.8) | 52 (24.6) | 0.931 |
| Barrett's oesophagus (enrolment) = Yes (%) | 1 (0.6) | 4 (2.0) | 0.48 |
| Barrett's oesophagus (follow-up) = Yes (%) | 4 (2.5) | 10 (5.3) | 0.288 |
| Symptom severity past 24 hours at enrolment (mean (SD)) | 1.83 (2.00) | 1.89 (2.15) | 0.785 |
| Symptom severity past 7 days at enrolment (mean (SD)) | 2.24 (2.03) | 2.28 (2.23) | 0.846 |
| Symptom severity past 30 days at enrolment (mean (SD)) | 2.46 (2.01) | 2.50 (2.27) | 0.878 |
| Symptom severity past 24 hours at last visit (mean (SD)) | 1.64 (1.89) | 1.60 (1.79) | 0.811 |
| Symptom severity past 7 days at last visit (mean (SD)) | 1.97 (1.95) | 1.69 (1.78) | 0.149 |
| Symptom severity past 30 days at last visit (mean (SD)) | 2.07 (2.01) | 1.80 (1.75) | 0.158 |
| Worst symptom severity in the last year (mean (SD)) | 2.42 (2.06) | 2.17 (1.95) | 0.234 |
| Any atopic disease = Yes (%) | 135 (76.3) | 169 (80.5) | 0.379 |
| Rhinoconjunctivitis = Yes (%) | 103 (58.2) | 139 (66.2) | 0.13 |
| Asthma = Yes (%) | 57 (32.2) | 71 (33.8) | 0.821 |
| Oral allergy syndrome = Yes (%) | 84 (47.5) | 89 (42.4) | 0.369 |
| Atopic dermatitis = Yes (%) | 26 (14.7) | 28 (13.3) | 0.813 |
| Nasal Polyposis = Yes (%) | 6 (3.4) | 7 (3.3) | 1.000 |

*Supplementary Table 5 Comparison of baseline characteristics, EoE symptom severities, and comorbidities of responders and non-responders to the additional CRS questionnaire.*

**Figure Legends**

Supplementary Figure S1 Marginal probabilities of high symptom severity stratified by CRS status at different time points. Asterisks’ indicate statistically significant differences (* for p < 0.05, ** for p < 0.01)
